# The impact of achievement emotions on learning performance in online learning context: a meta-analysis

**DOI:** 10.3389/fpsyg.2025.1599543

**Published:** 2025-07-21

**Authors:** Mengmeng Qi, Wenying Liu, Mingyue Li, Gaoge Wang, Bowen Liu

**Affiliations:** Faculty of Artificial Intelligence in Education, Central China Normal University, Wuhan, China

**Keywords:** online learning, achievement emotions, learning performance, meta-analysis, moderating effect

## Abstract

**Introduction:**

In online learning context, achievement emotions are of great significance and exert an influence on students’ learning performance. However, the research conclusions about the impact of achievement emotions on learning performance are not uniform.

**Methods:**

Following PRISMA guidelines, we searched Web of Science, Scopus, EBSCO and Google Scholar (2012–2024). 21 studies (240 independent effect sizes) met inclusion criteria and were analyzed with a random-effects model. Moreover, multiple moderating variables were investigated in the models, such as learner levels, disciplines, online learning styles, technological types, and types of performance measure.

**Results:**

Positive emotions showed a moderate positive correlation with online learning performance (r = 0.478), whereas negative emotions showed a moderate negative correlation (r = –0.303). Technology type and performance-measure type significantly moderated these relationships, while learner level, discipline, and online learning style did not.

**Discussion:**

Findings underscore the importance of fostering positive emotions in online learning and of adopting technologies that lessen the detrimental influence of negative emotions. Implications for instructional design and future research are discussed.

## 1 Introduction

The rapid advancement of information and communication technologies has led to a growing emphasis on online learning (Huang et al., 2023). The convenience and flexibility offered by online platforms have attracted an increasing number of students (Dumford and Miller, 2018; Mackavey and Cron, 2019; Zhang and Liu, 2019). Compared to traditional classroom settings, online learning environments tend to evoke more diverse and intense achievement-related emotions (Moneta and Kekkonen-Moneta, 2007; Lee and Chei, 2020). For instance, technical barriers or lack of interaction may increase frustration or boredom among students (Hamilton et al., 2023). During the COVID-19 pandemic, many students experienced heightened emotional fatigue, disengagement, and anxiety in online courses, largely due to inadequate instructional support and limited peer collaboration (Khan et al., 2021; Mahajan and Kalpana, 2018). It is essential to examine achievement emotions in online learning and understand their impact on learning performance.

Achievement emotions have been widely recognized as key determinants of educational performance. Drawing on Control-Value Theory (CVT), achievement emotions are defined as emotions directly related to achievement activities or outcomes, such as learning, task completion, or performance evaluation. Achievement emotions are understood to arise from learners’ appraisals of control (e.g., perceived competence) and value (e.g., task importance), and in turn shape cognitive engagement, motivation, and performance (Pekrun, 2006; Liu et al., 2022). Positive emotions, such as enjoyment and pride, tend to enhance students’ persistence, problem-solving, self-regulation, and satisfaction (Feng et al., 2023; Mega et al., 2014; Wu et al., 2021), while also promoting deeper processing and goal attainment (Luo and Luo, 2022; Lichtenfeld et al., 2023). In contrast, negative emotions, such as anxiety and boredom, can reduce cognitive capacity and attention allocation (Derakshan et al., 2009), impair motivation, and hinder task completion—effects often amplified in online learning environments due to higher demands for self-regulation and limited instructional support.

Nevertheless, empirical findings on the relationship between achievement emotions and academic performance in online learning remain inconsistent. While some studies report strong associations between emotions and performance outcomes (Pan et al., 2023; Zhu et al., 2022), others find little to no effect. For example, Wang et al. (2022) found no significant correlation between emotions and students’ online participation, and You and Kang (2014) observed negligible effects of anxiety and boredom on self-regulated learning. Variations in learning styles, instructional design, disciplinary norms, and assessment methods may account for these inconsistent findings (Dospinescu and Dospinescu, 2020; Tzafilkou et al., 2021). Consequently, a more targeted and rigorous synthesis is required to determine the specific conditions under which achievement emotions influence learning performance in online contexts.

Although prior research has examined the relationship between achievement emotions and learning performance, most meta-analyses have focused on general or traditional educational contexts, with limited attention paid to fully online learning environments. Moreover, the influence of specific moderating variables—such as learner level, discipline, online learning style, technology type, and performance measurement type—remains underexplored in the existing literature. These limitations hinder a nuanced understanding of how achievement emotions operate in technology-mediated settings. To address these gaps, the present study conducts a meta-analysis that focuses exclusively on online learning environments and systematically investigates how various contextual moderators shape the relationship between achievement emotions and learning performance. This study aims to provide a more targeted and context-sensitive synthesis that can inform both theoretical development and instructional practice in digital education.

## 2 Literature review

### 2.1 Achievement emotions

Achievement emotions refer to those feelings directly linked to the processes and outcomes of achievement-related activities (Pekrun, 2006), including emotions enjoyment, despair, anxiety, and hope. Pekrun’s Control-Value Theory (Pekrun and Linnenbrink-Garcia, 2012) highlights valence as a key factor in categorizing these emotions. According to this theory, achievement emotions can be divided into two main categories based on their valence: positive emotions (e.g., enjoyment, pride, relaxation) and negative emotions (e.g., anxiety, boredom, frustration). Positive emotions are typically associated with favorable experiences and outcomes, often leading to enhanced motivation and improved learning performance. In contrast, negative emotions may impair attention, reduce self-efficacy, and hinder performance.

### 2.2 Learning performance

To categorize learning performance in this study, we adopt Bloom’s Taxonomy of Educational Objectives, a widely recognized framework comprising three domains: cognitive, affective, and psychomotor (Bloom et al., 1956). The cognitive domain emphasizes the development of intellectual skills, progressing from basic knowledge recall to higher-order thinking such as analysis, synthesis, and evaluation (Krathwohl, 2002). These levels are interrelated and foundational to academic performance. The affective domain addresses emotional engagement with learning, including receiving, responding, valuing, organizing, and internalizing values. Emotions are not only outcomes but also drivers of deep cognitive processing, making them essential to meaningful learning (Sacks and Pikas, 2013). The psychomotor domain, though less frequently applied in general education research, concerns physical skills and coordinated actions. Simpson (1966) identified levels ranging from perception to innovation, emphasizing learners’ ability to adapt motor responses to environmental demands. This study adopts this framework to categorize students’ learning performance into three dimensions: cognitive outcomes, behavioral outcomes, and affective outcomes. This categorization provides a comprehensive and theory-driven basis for analyzing how achievement emotions relate to different facets of learning in online environments.

### 2.3 Achievement emotions and learning performance in online learning context

Achievement emotions, encompassing both positive and negative affective states, play a crucial role in shaping students’ learning performance in online learning environments. However, prior research presents mixed findings regarding their effects. Empirical studies largely support a positive association between emotions such as enjoyment and pride and online learning performance. Positive emotions have been linked to increased self-efficacy, engagement, and learning satisfaction (Artino and Jones, 2012; Heckel and Ringeisen, 2019; Wu et al., 2021). They also facilitate deep learning strategies and self-regulation (You and Kang, 2014; Yildiz Durak and Atman Uslu, 2023). Nonetheless, not all findings are consistent. Some studies suggest that emotions like relaxation and relief may reduce strategic engagement without directly affecting outcomes (Pekrun, 2017; Liu et al., 2021), while others report no significant link between enjoyment and performance (Parker et al., 2021). These results imply that the effects of positive emotions may depend on emotion type, learning context, or task characteristics.

Negative emotions such as anxiety, boredom, and frustration are typically associated with reduced engagement, lower satisfaction, and poorer performance in online settings (Tzafilkou et al., 2021; Lai et al., 2021). They often impair cognitive resources and hinder sustained attention (Cheng et al., 2023; Heckel and Ringeisen, 2019). However, several studies suggest that moderate levels of negative emotion can promote effort and persistence. For example, anxiety has been found to enhance self-regulatory behaviors in some contexts (Yildiz Durak and Atman Uslu, 2023), and frustration may serve as a motivational trigger in collaborative learning or MOOC environments (Hilliard et al., 2020; Liu et al., 2021).

### 2.4 Potential moderators of the relationship between achievement emotions and learning performance

Variations in research findings on achievement emotions and learning performance may be attributed to sample characteristics and methodological differences. To account for such heterogeneity, this study examines five potential moderators: *learner level, discipline, online learning style, technology type, and type of performance measure*.

*Learner level* refers to the formal stage of schooling of the sample and is coded in three mutually categories: primary, secondary and tertiary. Learner level may influence how achievement emotions relate to performance. Research shows stronger emotion-performance links among secondary students compared to primary or tertiary learners (Camacho-Morles et al., 2021). This may reflect developmental shifts—positive emotions tend to decline and negative ones rise during adolescence (Meyer and Schlesier, 2022; Ma and Kishor, 1997). When positive feedback is dominant, motivation and self-efficacy improve, strengthening emotional impacts on learning (Pekrun et al., 2017).

*Discipline* distinguishes the knowledge domain of a course: humanities and social sciences, and natural sciences. Discipline can shape how students perceive and react emotionally to learning tasks. According to Control-Value Theory, subject characteristics affect emotional value appraisal (Pekrun, 2006). Emotions like anxiety show weaker effects in language subjects than in mathematics, while hopelessness is less detrimental in English than in math (Goetz et al., 2013).

*Online learning style* refers to the structural modality through which instructional content and interactions are organized in digital environments. Based on existing classifications in online education research (Fabriz et al., 2021), we categorize online learning styles into three types: synchronous, asynchronous, and blended. Online synchronous learning refers to real-time, simultaneous interactions among learners and instructors (e.g., live lectures, virtual classrooms). Online asynchronous learning allows learners to access instructional materials and complete tasks at their own pace without real-time constraints (e.g., pre-recorded lectures, discussion boards). Blended learning integrates both synchronous and asynchronous elements within the same learning experience. Online learning style may influence how achievement emotions affect learning. In asynchronous settings, positive emotions promote persistence and improve performance (Yu et al., 2020). However, in synchronous formats, this link appears weaker or non-significant (Apridayani et al., 2023), suggesting that interaction structure and immediacy may shape emotional dynamics differently.

*Technology type* refers to the category of digital tools or platforms used to support online learning, which may shape learners’ emotional experiences and cognitive engagement. In this study, we differentiate five categories: information-access technologies, communication and collaboration technologies, efficiency-oriented technologies, cognitive and situational technologies, and commenting and other technologies. Information-access technologies include tools primarily designed to deliver instructional content and provide access to learning materials. Communication and collaboration technologies refer to platforms that facilitate social interaction, peer dialog, and group-based learning. Efficiency-oriented technologies encompass systems aimed at improving learning efficiency, task completion, and personalized feedback. Cognitive and situational technologies engage learners in active meaning-making and immersive learning experiences. Commenting and other technologies include tools that allow learners and instructors to annotate, mark up, or provide feedback on digital content. Technology type may moderate emotional influences by shaping learners’ cognitive and affective experiences. According to Affective Events Theory, different technologies elicit distinct emotional responses that influence behavior over time (Weiss and Beal, 2005). Research shows that digital tools can reduce boredom, increase enjoyment, and enhance engagement, particularly in STEM contexts (Loderer et al., 2020; Stilin et al., 2023).

*The type of performance measure* refers to the method by which students’ learning outcomes were assessed in the included primary studies. Given that different assessment methods may capture distinct dimensions of performance and be differentially sensitive to emotional influences, this variable was treated as a potential moderator. In this study, it is categorized as self-reported measures, standardized tests, and system automatic recordings. Self-reported measures refer to students’ subjective evaluations of their learning performance, often collected through questionnaires or rating scales. Standardized tests involve formal, externally developed instruments designed to assess academic achievement in a structured and often high-stakes format. System automatic recordings include automatically recorded behavioral indicators from digital platforms. Type of performance measure may influence the observed strength of emotion–performance associations. Emotions tend to show stronger effects when outcomes are assessed through standardized tests closely tied to academic success (Camacho-Morles et al., 2021). In contrast, effects are often weaker with self-reports or subjective evaluations, which may reflect broader perceptions rather than direct performance.

### 2.5 Research hypotheses and conceptual model

**Figure 1:**
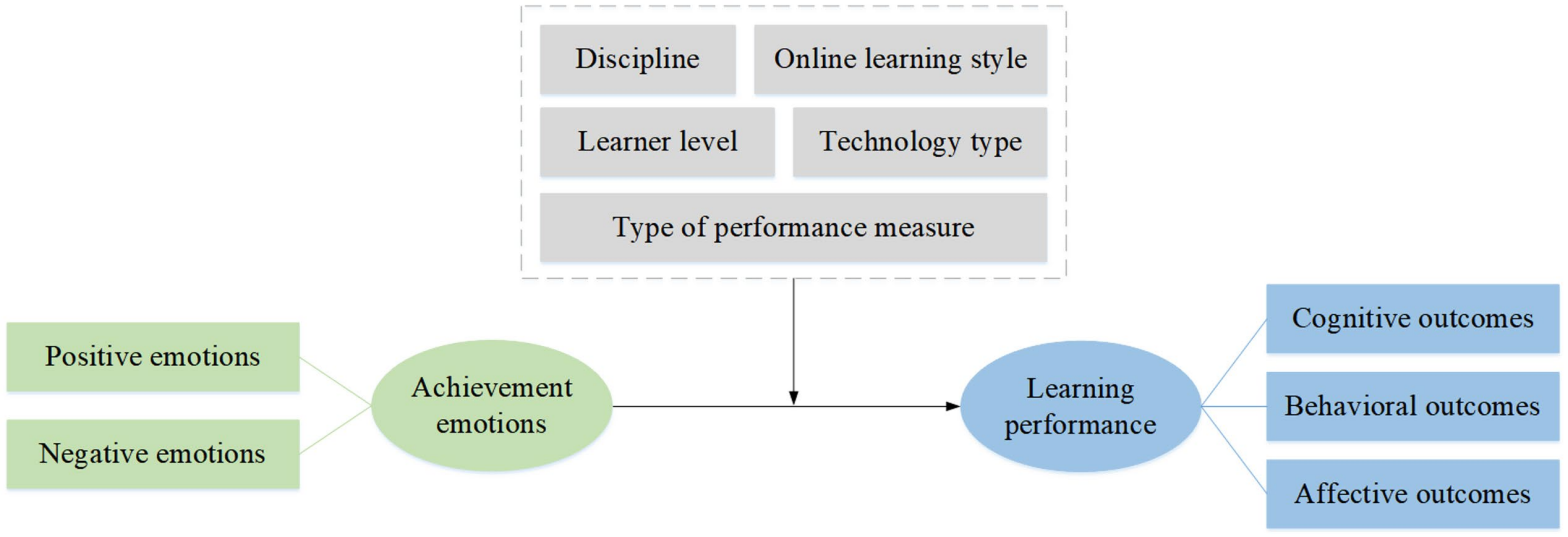
The research model.

This study intends to investigate the relationships between achievement emotions and learning performance within online learning environments. We present a research model to clarify how students’ emotional experiences affect their learning performance in online learning contexts (see Figure 1). Drawing on Control-Value Theory (Pekrun, 2006) and previous empirical findings, the following hypotheses are proposed:

*H1:* Positive emotions are positively correlated with learning performance in online learning context.

*H2:* Negative emotions are negatively correlated with learning performance in online learning context.

*H3:* Learner level moderates the relationship between achievement emotions and learning performance in online learning context.

*H4:* Discipline moderates the relationship between achievement emotions and learning performance in online learning context.

*H5:* Online learning style moderates the relationship between achievement emotions and learning performance in online learning context.

*H6:* Technology type moderates the relationship between achievement emotions and learning performance in online learning context.

*H7:* Type of performance measure moderates the relationship between achievement emotions and learning performance in online learning context.

### 2.6 Prior systematic reviews and meta-analysis

Several meta-analytic studies have examined the relationship between achievement emotions and learning performance. Lei and Cui (2016) found that positive emotions were positively associated with academic achievement, while negative emotions showed adverse effects in studies from mainland China. Camacho-Morles et al. (2021) confirmed similar patterns across specific emotions, with enjoyment positively linked to performance, and anger and boredom having negative associations; frustration, however, showed minimal impact. In the domain of language learning, He et al. (2024) reported a moderate positive correlation between enjoyment and performance, and a moderate negative correlation for anxiety and boredom. Huang (2011) demonstrated that achievement emotions are closely tied to goal orientation, with negative emotions associated with avoidance goals and positive emotions linked to relational goals. In technology-based learning environments, Loderer et al. (2020) reported a small to moderate positive effect of enjoyment (*r* = 0.18), while other negative emotions had minimal influence (e.g., anger, frustration, boredom all below *r* = −0.08).

Despite these advances, prior meta-analyses have largely focused on traditional or blended learning environments and often failed to consider the distinct emotional dynamics of fully online learning. Moreover, many studies emphasized specific subjects or emotion types, limiting generalizability. To address these gaps, the present study offers a comprehensive synthesis focusing exclusively on online contexts, incorporating the most frequently examined achievement emotions and a broader categorization of learning performance (cognitive, behavioral, and affective). As online learning continues to expand, this meta-analysis provides timely and context-specific insights for future research and practice.

## 3 Methods

### 3.1 Research process

The study selection process and eligibility criteria were developed and applied in accordance with established meta-analytic procedures (Borenstein et al., 2009; Chang et al., 2022), ensuring methodological rigor and replicability. The meta-analytic process carried out in this study consisted of the following steps: (1) defining the research objectives; (2) conducting a systematic literature search and applying predefined inclusion and exclusion criteria; (3) coding the eligible studies and extracting effect sizes; (4) selecting an appropriate analysis model based on the heterogeneity test results; and (5) calculating the overall effect size and conducting main effect tests, publication bias analysis, and moderator analyses. The selection of studies followed a rigorous screening protocol, and all eligible studies were independently coded by two researchers. Comprehensive Meta-Analysis 2.0 (CMA 2.0) software was used to conduct the meta-analysis in this study.

### 3.2 Literature search strategy

To conduct a comprehensive literature review, this study primarily utilized Web of Science, Scopus, and EBSCO as the main databases for retrieving relevant articles, complemented by a search through Google Scholar for additional sources. A citation tracking method was also employed to identify further studies of interest. The literature search covered publications from January 2012 to September 2024. We used keyword combinations including “academic emotions,” “achievement emotions,” “positive emotions,” “negative emotions,” and specific emotion terms (e.g., anxiety, enjoyment, boredom) combined with “online learning” or “e-learning.” Only peer-reviewed journal articles published in English were considered. In addition to database searches, backward reference checking and citation chaining were used to identify additional studies.

### 3.3 Inclusion/exclusion criteria

The criteria for literature screening included the following: (1) the study examined the relationship between at least one achievement emotion and learning performance, with learning performance as the dependent variable; (2) the independent variable was an achievement emotion, not used as a moderator; (3) emotions were measured as discrete emotional states (e.g., self-reports or observational coding), excluding physiological or implicit measures; (4) the study context was a fully online learning environment; (5) sufficient statistical data (e.g., r values or convertible data) were provided to calculate effect sizes; and (6) the study was an empirical, peer-reviewed journal article. Studies such as literature reviews, theoretical papers, conference abstracts, or studies conducted in blended or face-to-face settings were excluded.

### 3.4 Selection strategy

**Figure 2:**
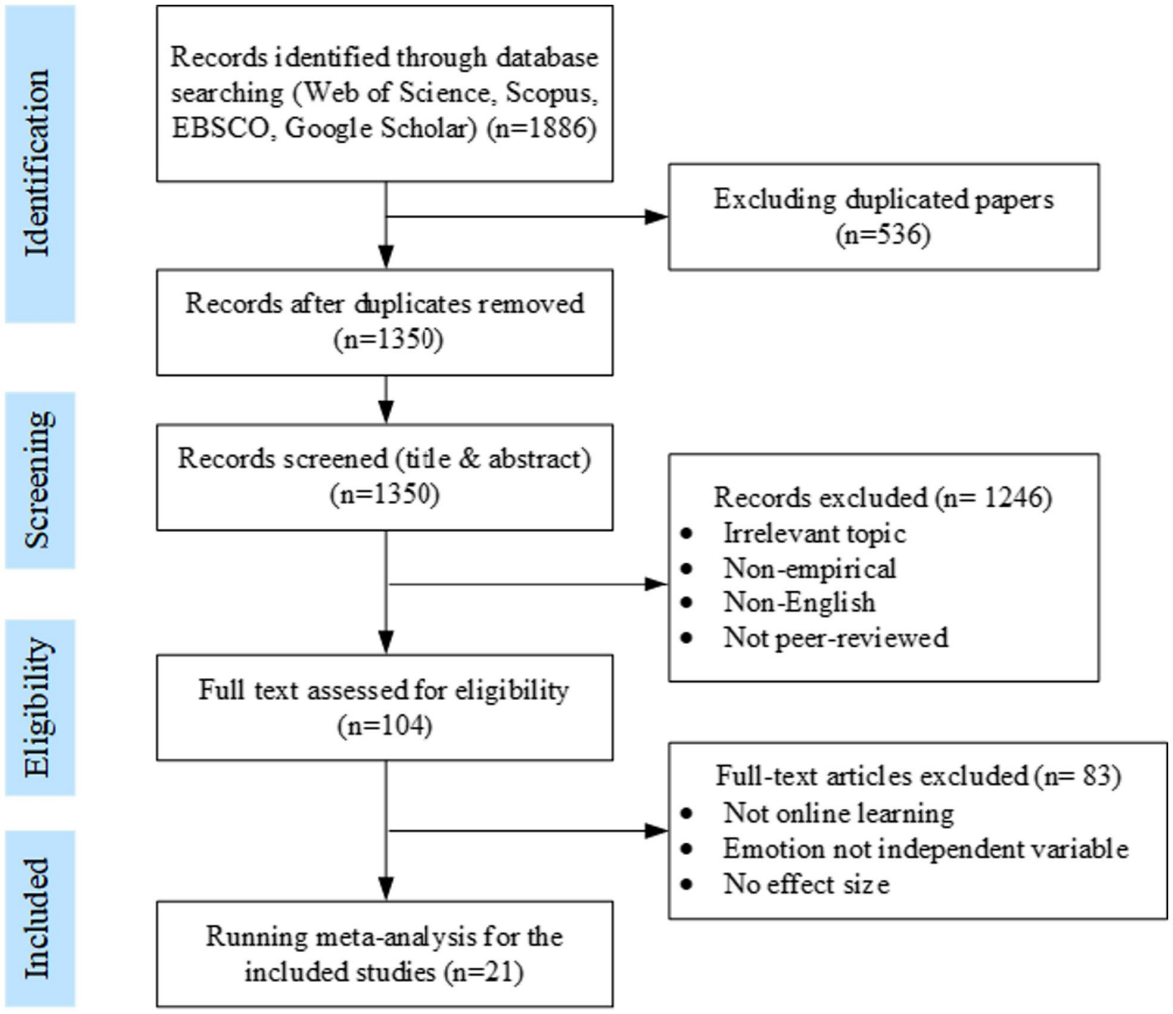
The process for selection of studies included in meta-analysis.

A total of 1886 records were initially identified through database searches. After removing duplicates, 1,350 unique records remained. The study selection proceeded in three stages. First, titles and abstracts were screened to remove unrelated topics and non-empirical studies, resulting in 104 potentially eligible articles. Second, full-text screening was conducted using the inclusion and exclusion criteria. Finally, 21 studies were retained for the meta-analysis. The selection process is summarized in Figure 2.

To ensure strong alignment with the research objectives, only studies conducted in fully online learning environments were included. These covered diverse modalities (e.g., synchronous, asynchronous) and technology types (e.g., communication and collaboration tools, efficiency-oriented tools), reflecting the current landscape of digital learning. Participants spanned secondary and tertiary education levels, and represented both humanities/social sciences and natural sciences, ensuring broad demographic and disciplinary coverage. This diversity enhances the generalizability of findings across learner types and contexts. All included studies reported key statistical indicators (e.g., effect sizes, sample sizes, correlations) and used varied performance assessments (e.g., standardized tests, self-reports, system logs). Studies failing to meet methodological rigor were excluded to ensure sample reliability.

### 3.5 Coding

Following the selection strategy, all eligible studies were independently coded by two researchers. The standardized correlation coefficient (r) was chosen as the statistical effect size in this study. The computational process is a two-stage process that first requires a Fisher’s Z transformation of the sample correlation coefficients and a meta-analysis using the transformed values, followed by a further transformation of the combined effect values into correlation coefficients. The coded variables in this study included the type of achievement emotions, learning performance, learner level, discipline, online learning style, technology type and type of performance measure (Table 1). Based on the control-value theory, achievement emotions were categorized as positive emotions (happy, hopeful, proud…etc.), negative emotions (anxious, angry, ashamed, helpless, bored, frustrated, disappointed…etc.). Learning performance was categorized into three main groups: cognitive, behavioral, and affective outcomes. The cognitive outcomes included GPA, test scores, learning achievement, self-regulation…etc. The behavioral outcomes referred to technology use, learning interaction, learning engagement…etc. The affective outcomes referred to learning attitude, learning motivation, learning interest, learning satisfaction, self-efficacy, academic procrastination, intention to persist…etc.

**Table 1:** Coding information for meta-analysis.

Categories	Coding information
Achievement emotions	Positive emotions, negative emotions.
Learning performance	Cognitive, behavioral, and affective domains.
Learner level	Tertiary, secondary, and primary.
Discipline	Humanities and social sciences, and natural sciences.
Online learning style	Online synchronous learning, online asynchronous learning, blended learning (online synchronous learning & online asynchronous learning).
Technology type	Information access technologies, communication and collaboration technologies, efficiency-oriented technologies, cognitive and situational technologies, and commenting and other technologies.
Type of performance measure	Self-reported measures, standardized tests and system automatic recording.
Others	Descriptive data: title, author, year, countries of study.

Learner levels were divided into: tertiary (undergraduate and graduate students), secondary (grades 7–12), and primary (grades 1–6); The disciplines were divided into: humanities and social sciences (e.g., Language Arts, History, English, etc.), and natural sciences (e.g., math, physics, biology, chemistry, etc.). The online learning styles were divided into online synchronous learning, online asynchronous learning and blended learning (online synchronous learning & online asynchronous learning). The technology types were divided into information access technologies (e.g., web, mobile devices, computers, etc.), communication and collaboration technologies (e.g., QQ, WeChat, Tencent conference, etc.), efficiency-oriented technologies (e.g., PPT, Flash, electronic whiteboards, web-based platforms, etc.), cognitive and situational technologies (e.g., mind mapping, virtual environments, etc.), and commenting and other technologies (e.g., robots, voting systems, etc.). Types of performance measure were divided into: self-reported measures and standardized tests (e.g., tests used in comparable studies, or more general tests such as national exams). The samples were coded by two researchers, demonstrating a Kappa coefficient of 0.937, with a high consistency (Card, 2012). Any disagreements in coding were addressed through discussion until consensus was reached. The coding of the 21 studies is shown in the appendix. We have uploaded the full dataset used in this meta-analysis to FigShare (10.6084/m9.figshare.29391194.).

### 3.6 Data analysis

In line with the meta-analysis methodology expounded by Lipsey and Wilson (2001), correlation coefficients were deployed to assess the extent of the influence while delving into the connection between achievement emotions and learning performance. Initially, Spearman’s rank-order and regression correlation coefficients underwent a transformation into Pearson correlation coefficients, with the objective of homogenizing the effect magnitude throughout the research endeavor. Subsequently, heeding the counsel of Hedges and Olkin (1985), all the correlation coefficients were transmuted into Fisher’s Z-scores to mollify the variance. For the purpose of facilitating comprehension, these Z-scores were subsequently reconverted to Pearson correlation coefficients. In accordance with the normative benchmarks proposed by Cohen (1992), effect sizes were categorized, with values of 0.2, 0.5, and 0.8 being indicative of small, moderate, and large magnitudes, respectively. Upon the completion of all analyses, results were reported only when the quantity of studies reached a threshold of k ≥ 5, thereby providing a more robust foundation for interpretation, as recommended by Lipsey and Wilson (2001).

## 4 Results

### 4.1 Publication Bias

**Figure 3:**
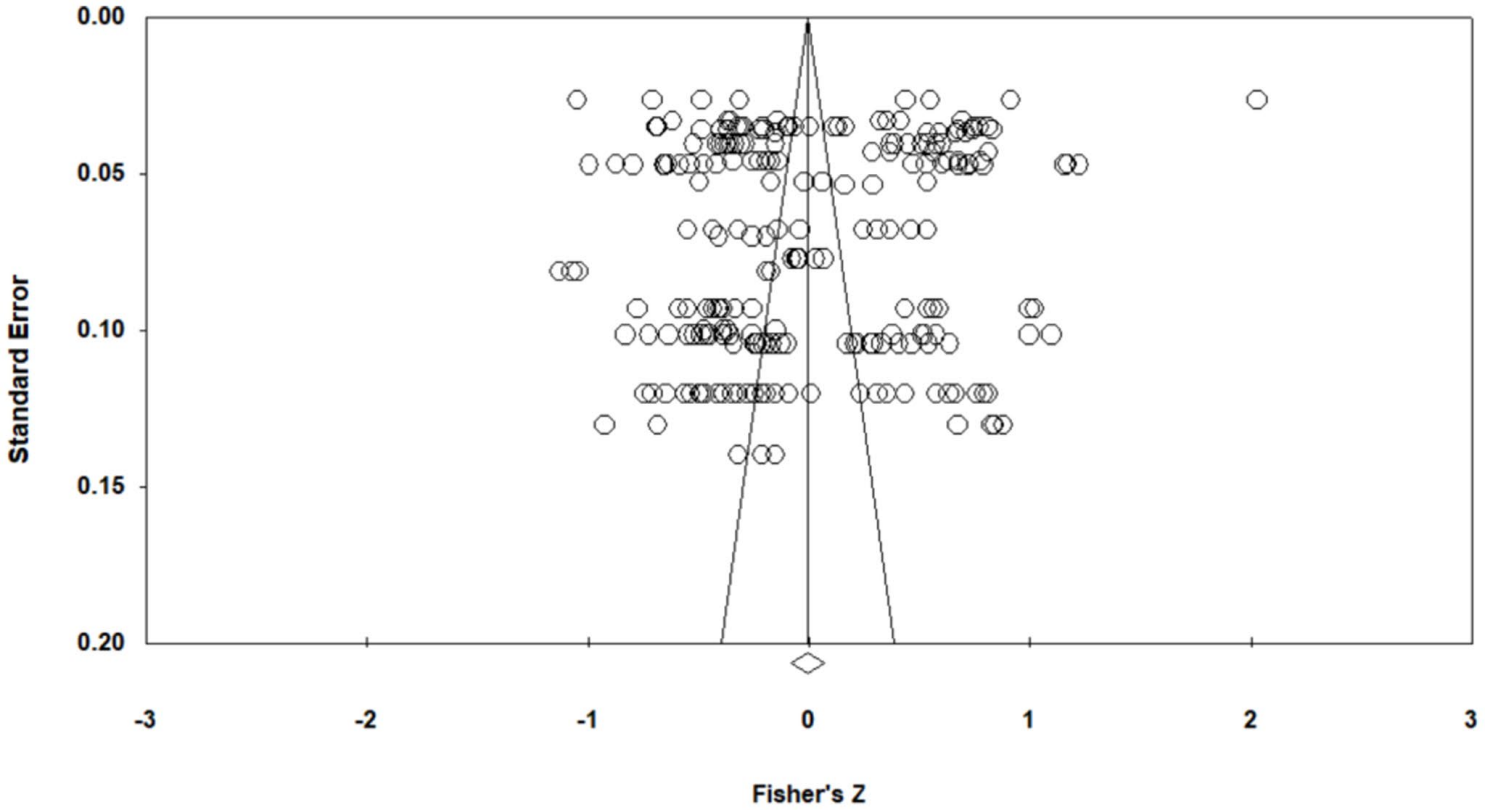
Funnel plot of effect sizes of the correlation between achievement emotions and learning performance.

To assess the potential existence of publication bias, several methods were used, including a qualitative funnel plot, Egger’s test, and Rosenthal’s Fail-safe N method. When the distribution of impact magnitude shows symmetry close to the mean, it suggests that the likelihood of publication bias is quite low. The funnel plot reveals that the effect size scatter points of most studies are evenly and symmetrically distributed around the average effect size, indicating a low probability of publication bias (see Figure 3). The Rosenthal’s Nfs is 2862, which is greater than 1,210 (5*240 + 10) and much greater than 5 K + 10 (k refers to the number of independent effect sizes included in the meta-analysis), *p* < 0.001. This indicates that the study sample literature was relatively unaffected by publication bias, further demonstrating the absence of publication bias. The results of Egger’s test showed that, t = 1.476 < 1.96, *p* = 0.141 > 0.05, further evidence of the absence of publication bias.

### 4.2 Heterogeneity test

Table 2 provides the results of the heterogeneity analysis for the studies incorporated within this meta-analysis. The overall Q statistic was 30,657.606 (*p* < 0.001), exceeding the degrees of freedom (df = 239), which suggests substantial heterogeneity among the studies. The I^2^ statistic was 99.22%, significantly higher than the 75% threshold, indicating a very high degree of variability in the effect sizes. This implies that 99.22% of observed variability can be attributed to actual differences in the degree of impact between studies, rather than sampling error. In view of these revelations, a stochastic effect model was utilized to appraise the overall effect size, as recommended by Lipsey and Wilson (2001). Additionally, a moderator analysis was conducted to probe into the potential sources responsible for this observed heterogeneity, with the aim of enhancing the understanding and interpretation of the meta-analysis results.

**Table 2:** The impact of achievement emotions on learning performance.

Effect model	Sample size	*r*	95% CI		Heterogeneity test			
			Lower	Upper	Q	df	*p*	*I*^2^
Fixed effect model	240	0.054	0.048	0.061	30657.606	239	0.000	99.220
Random effects model	240	−0.007	−0.082	0.068				

### 4.3 Main effects analysis

This study analyzed 240 independent effect sizes to explore the relationship between achievement emotions and learning performance. Following Cohen (1992) criteria, effect sizes around 0.2 are considered small, approximately 0.5 moderate, and close to 0.8 large.

The results of the principal effects analysis, presented in Table 3, reveal that positive emotions are moderately positively associated with learning performance (*r* = 0.478), while negative emotions are moderately negatively associated (*r* = −0.303). Specifically, in terms of affective outcomes, positive emotions show a moderate positive correlation with learning performance (*r* = 0.462), whereas negative emotions exhibit a moderate negative correlation (*r* = −0.223). For behavioral outcomes, positive emotions are moderately positively correlated (*r* = 0.616), while negative emotions are moderately negatively correlated (*r* = −0.410). Regarding cognitive outcomes, positive emotions are also moderately positively related to learning performance (*r* = 0.474), while negative emotions demonstrate a moderate negative correlation (*r* = −0.294).

**Table 3:** Main effects analysis for the relationship between achievement emotions and learning performance.

Achievement emotions	Learning performance	*k*	*r*	95%CI		Two-tailed test	
				Lower	Upper	*Z*	*p*
Positive emotions	Affective outcomes	33	0.462	0.361	0.552	8.027	0.000
	Behavioral outcomes	17	0.616	0.376	0.778	4.363	0.000
	Cognitive outcomes	33	0.474	0.398	0.544	10.748	0.000
	Overall	83	0.478	0.420	0.532	14.050	0.000
Negative emotions	Affective outcomes	53	−0.223	−0.355	−0.083	−3.097	0.002
	Behavioral outcomes	28	−0.410	−0.507	−0.302	−6.897	0.000
	Cognitive outcomes	76	−0.294	−0.341	−0.246	−11.383	0.000
	Overall	157	−0.303	−0.344	−0.261	−13.471	0.000

*k*, number of independent studies; *r*, effect size; 95% CI, 95% confidence interval; *Z*, Fisher’s Z transformation results; *p*, significance level.

### 4.4 Moderating effect analysis

This study performed subgroup analysis for categorical variables. For the effect size across different learner levels, the test for inter-group differences in the effect of positive emotions did not reach statistical significance (Q_B_ = 1.366, *p* = 0.513 > 0.05), indicating no significant variation in the impact of positive emotions on learning performance across learner levels. However, a trend was observed where secondary learners showed the highest effect (*r* = 0.531, 95% CI [0.331, 0.685], *p* < 0.001), followed by tertiary (*r* = 0.500) and combined groups (*r* = 0.418), all indicating moderate positive effects. For negative emotions, the inter-group effect test also did not reveal statistical significance (Q_B_ = 0.246, *p* = 0.884 > 0.05), suggesting no significant differences in the effect of negative emotions on learning performance across learner levels. Nevertheless, the largest effect was found in the combined group (*r* = −0.324), with slightly smaller effects in tertiary (*r* = −0.298) and secondary learners (*r* = −0.268), all reflecting small but significant negative associations. These patterns suggest that while differences were not statistically significant, younger students may experience stronger effects from achievement emotions on learning performance (see Table 4).

**Table 4:** Effects of learning performance on the types of learner levels.

Achievement emotions	Moderator	*k*	*r*	*z*	95%CI		Q_B_	*p* value
					LL	UL		
Positive emotions	Learner level						1.366	0.513
	Tertiary	63	0.500	9.570^***^	0.411	0.579		
	Secondary	16	0.531	4.679^***^	0.331	0.685		
	Tertiary and secondary	4	0.418	5.242^***^	0.272	0.545		
Negative emotions	Learner level						0.246	0.884
	Tertiary	116	−0.298	−9.426^***^	−0.355	−0.239		
	Secondary	34	−0.268	−2.426^*^	−0.46	−0.053		
	Tertiary and secondary	7	−0.324	−4.848^***^	−0.44	−0.198		

*k*, number of independent studies; *r*, effect size; 95% CI, 95% confidence interval; Q_B_, between-group homogeneity; ^*^p < 0.05, ^**^p < 0.01, ^***^p < 0.001.

For the effect sizes across disciplines, positive emotions showed the highest effect in the humanities and social sciences (*r* = 0.529, 95% CI [0.417, 0.626], *p* < 0.001), followed by the “unknown” category (*r* = 0.515, 95% CI [0.385, 0.624], *p* < 0.001) and natural sciences (*r* = 0.401, 95% CI [0.300, 0.493], *p* < 0.001). However, the group effect test results (Q_B_ = 3.730, *p* = 0.155 > 0.05) indicate no significant differences in the effect of positive emotions on learning performance across disciplines. For negative emotions, the strongest effect was observed in the natural sciences (*r* = −0.352, 95% CI [−0.428, −0.272], *p* < 0.001), followed by the humanities and social sciences (*r* = −0.311, 95% CI [−0.373, −0.246], *p* < 0.001) and the unknown category (*r* = −0.208, 95% CI [−0.343, −0.065], *p* < 0.001). Similarly, the group effect test (Q_B_ = 3.232, *p* = 0.199 > 0.05) revealed no significant differences in the effect of negative emotions on learning performance across disciplines. Overall, while the group differences were not statistically significant, the pattern suggests that emotional influences may be somewhat more pronounced in the humanities and social sciences for positive emotions, and in the natural sciences for negative emotions (see Table 5).

**Table 5:** Effects of learning performance on the types of disciplines.

Achievement emotions	Moderator	*k*	*r*	*z*	95%CI		Q_B_	*p* value
					LL	UL		
Positive emotions	Discipline						3.730	0.155
	Humanities and social sciences	44	0.529	7.945^***^	0.417	0.626		
	Unknown	22	0.515	6.844^***^	0.385	0.624		
	Natural sciences	17	0.401	7.231^***^	0.300	0.493		
Negative emotions	Discipline						3.232	0.199
	Humanities and social sciences	72	−0.311	−8.939^***^	−0.373	−0.246		
	Unknown	44	−0.208	−2.837^**^	−0.343	−0.065		
	Natural sciences	41	−0.352	−8.058^***^	−0.428	−0.272		

*k*, number of independent studies; *r*, effect size; 95% CI, 95% confidence interval; Q_B_, between-group homogeneity; ^*^p < 0.05, ^**^p < 0.01, ^***^p < 0.001.

For the effect size of each online learning style, as for positive emotions, effect values were higher for online synchronous learning & online asynchronous learning (*r* = 0.624, 95% CI [0.371, 0.791], *p* < 0.001) and unknown (*r* = 0.511, 95% CI [0.401, 0.607], *p* < 0.001), followed by online synchronous learning (*r* = 0.492, 95% CI [0.365, 0.601], *p* < 0.001) and online asynchronous learning (*r* = 0.412, 95% CI [0.317, 0.499], *p* < 0.001). Overall, from the test results of group effects (Q_B_ = 3.951, *p* = 0.267 > 0.05), there is no significant difference in the influence of positive emotions on learning performance under different online learning styles. As for negative emotions, effect values were higher for online synchronous learning & online asynchronous learning (*r* = −0.371, 95% CI [−0.530, −0.187], *p* < 0.001) and online synchronous learning (*r* = −0.320, 95% CI [−0.386, −0.250], *p* < 0.001), followed by unknown (*r* = −0.280, 95% CI [−0.387, −0.166], *p* < 0.001) and online asynchronous learning (*r* = −0.259, 95% CI [−0.317, −0.198], *p* < 0.001). Overall, from the test results of group effects (Q_B_ = 2.626, *p* = 0.453 > 0.05), there is no significant difference in the impact of negative emotions on learning performance in different online learning styles. While the statistical differences were nonsignificant, the observed effect patterns suggest potential differences in emotional impact across online learning formats (see Table 6).

**Table 6:** Effects of learning performance on the types of online learning styles.

Achievement emotions	Moderator	*k*	*r*	*z*	95%CI		Q_B_	*p* value
					LL	UL		
Positive emotions	Online learning style						3.951	0.267
	Unknown	33	0.511	7.925^***^	0.401	0.607		
	Online synchronous learning	21	0.492	6.747^***^	0.365	0.601		
	Blended learning	11	0.624	4.192^***^	0.371	0.791		
	Online asynchronous learning	18	0.412	7.813^***^	0.317	0.499		
Negative emotions	Online learning style						2.626	0.453
	Unknown	73	−0.280	−4.685^***^	−0.387	−0.166		
	Online synchronous learning	47	−0.320	−8.575^***^	−0.386	−0.250		
	Blended learning	10	−0.371	−3.812^***^	−0.530	−0.187		
	Online asynchronous learning	27	−0.259	−8.148^***^	−0.317	−0.198		

*k*, number of independent studies; *r*, effect size; 95% CI, 95% confidence interval; Q_B_, between-group homogeneity; ^*^p < 0.05, ^**^p < 0.01, ^***^p < 0.001.

Regarding technology types, positive emotions had the strongest association with learning performance when mixed technologies were used (*r* = 0.596, 95% CI [0.530, 0.655], *p* < 0.001), followed by efficiency-oriented technologies (*r* = 0.484, 95% CI [0.346, 0.602], p < 0.001). However, the test for group differences did not reach significance (QB = 5.368, *p* = 0.068), suggesting no statistically significant variation across technology types. Technology type significantly moderated the relationship between negative emotions and learning performance (QB = 92.261, *p* < 0.001). Mixed technologies (*r* = −0.393, 95% CI [−0.446, −0.338]) and efficiency-oriented tools (*r* = −0.318, 95% CI [−0.400, −0.231]) exhibited stronger negative effects, while communication and collaboration tools showed a negligible and non-significant effect (*r* = −0.028, 95% CI [−0.081, 0.026], *p* = 0.311). This suggests that emotionally charged learning may be more disruptive in performance-focused platforms than in those centered on interaction or support (see Table 7).

**Table 7:** Effects of learning performance on the types of technologies.

Achievement emotions	Moderator	*k*	*r*	*z*	95%CI		Q_B_	*p* value
					LL	UL		
Positive emotions	Technology type						5.368	0.068
	Unknown	35	0.475	7.754^***^	0.367	0.569		
	Efficiency-oriented technologies	33	0.484	6.186^***^	0.346	0.602		
	Mixed technologies	15	0.596	13.898^***^	0.530	0.655		
Negative emotions	Technology type						92.261^***^	0.000
	Communication and collaboration technologies	8	−0.028	−1.014	−0.081	0.026		
	Unknown	86	−0.269	−5.563^***^	−0.356	−0.176		
	Efficiency-oriented technologies	33	−0.318	−6.850^***^	−0.400	−0.231		
	Mixed technologies	30	−0.393	−12.760^***^	−0.446	−0.338		

*k*, number of independent studies; *r*, effect size; 95% CI, 95% confidence interval; Q_B_, between-group homogeneity; ^*^p < 0.05, ^**^p < 0.01, ^***^p < 0.001.

The type of performance measurement significantly moderated the relationship between achievement emotions and learning performance. For positive emotions, self-reported measures (*r* = 0.522, 95% CI [0.447, 0.590], *p* < 0.001) yielded stronger associations than standardized tests (*r* = 0.355, 95% CI [0.221, 0.475], *p* < 0.001), while the effect for system automatic recording was weaker and non-significant (*r* = 0.256, 95% CI [−0.070, 0.532], *p* = 0.122). The intergroup test was statistically significant (QB = 7.480, *p* = 0.024), suggesting meaningful variation across performance assessment types. For negative emotions, self-reported measures (*r* = −0.318, 95% CI [−0.381, −0.252], *p* < 0.001) also showed a substantially stronger effect than standardized tests (*r* = −0.101, 95% CI [−0.155, −0.047], *p* < 0.001), whereas system automatic recording did not produce significant results (*r* = −0.333, 95% CI [−0.603, 0.005], *p* = 0.054). The intergroup effect test was significant (QB = 25.584, *p* < 0.001), reinforcing that the observed effect sizes depend considerably on how learning performance is measured (see Table 8).

**Table 8:** Effects of learning performance on the types of performance measures.

Achievement emotions	Moderator	*k*	*r*	*z*	95%CI		Q_B_	*p* value
					LL	UL		
Positive emotions	Type of performance measure						7.480^*^	0.024
	Self-reported measures	72	0.522	11.500^***^	0.447	0.590		
	Standardized tests	8	0.355	4.980^***^	0.221	0.475		
	System automatic recording	3	0.256	1.547	−0.070	0.532		
Negative emotions	Type of performance measure						25.584^***^	0.000
	Self-reported measures	133	−0.318	−9.038^***^	−0.381	−0.252		
	Standardized tests	21	−0.101	−3.677^***^	−0.155	−0.047		
	System automatic recording	3	−0.333	−1.93	−0.603	0.005		

*k*, number of independent studies; *r*, effect size; 95% CI, 95% confidence interval; Q_B_, between-group homogeneity; ^*^p < 0.05, ^**^p < 0.01, ^***^p < 0.001.

## 5 Discussion

### 5.1 The relationship between achievement emotions and learning performance in online learning context

This meta-analysis revealed that positive emotions exerted a moderate positive effect on students’ learning performance in online settings (*r* = 0.506), while negative emotions had a small-to-moderate negative impact (*r* = −0.313). These findings are consistent with theoretical frameworks suggesting that achievement emotions play a meaningful role in shaping learning performance (Pekrun et al., 2017).

Positive emotions not only broaden students’ attention, cognition, and memory but also facilitate their ability to acquire and process various learning resources, which helps students adopt more effective learning methods and strategies (Fredrickson and Branigan, 2005), leading to high performance. Positive emotions could facilitate the engagement of online learners, encouraging them to actively participate in learning activities and interact with content, peers and instructors. These interactions not only contribute to knowledge building, but also to reflective thinking about learning experiences and support the development of community awareness and self-regulating skills (Wang et al., 2022). Consequently, in the online learning environment, positive emotions are associated with heightened motivation, increased engagement, greater satisfaction, improved performance, and enhanced academic achievement. The broaden-and-build theory of positive emotions theorizes that “positive emotions expand learners’ attention and cognitive range” (Fredrickson, 2001; Fredrickson and Joiner, 2002). Given that attention and cognition are vital elements in the learning process, when positive emotions widen and augment students’ attention and cognitive focus toward learning, it subsequently leads to an elevation in academic performance. Positive emotions may enhance students’ attention and concentration levels, and stimulate their active participation in learning activities (Fredrickson, 2001). Moreover, positive emotions not only broaden the range of students’ attention, cognition, and memory, but also assist them in accessing and processing a diverse array of learning resources, thereby enabling them to adopt more optimal learning methods and strategies (Fredrickson and Branigan, 2005). When students are in positive emotions states, students will have a higher desire to learn, be more motivated to learn new knowledge, and be able to solve more problems when they have more knowledge, resulting in a better learning performance (Pekrun et al., 2017).

Negative emotions could weaken motivation (Sutter-Brandenberger et al., 2018), limit students’ cognitive resources, and hinder students’ learning engagement (Wang et al., 2022), leading to low performance. When students experience negative emotions, their attention is drawn to the cause of these feelings, diverting cognitive resources away from learning materials and toward distracting events or situations. Consequently, negative emotions may disrupt academic activities by reducing the resources essential for integrating and recalling key details (Valiente et al., 2012). Anxiety and anger could both impair students’ ability to recall relevant material (Linnenbrink, 2007). When faced with high levels of stress or negative emotions, cognitive functions such as memory retrieval are often compromised. This may lead to difficulties during exams or important presentations where recalling information is crucial. Negative emotions may lead students to pay more attention to difficulties in learning, thus limiting their access to the cognitive resources they need in their learning activities (Derakshan et al., 2009).

### 5.2 The moderating effects on the relationship between achievement emotions and learning performance in online learning context

The moderator analysis revealed notable variation in the strength of the relationship between achievement emotions and learning performance across studies. Among the five examined moderators—*learner level, discipline, online learning style, technology type, and type of performance measure*—technology type and type of performance measure demonstrated more pronounced moderating effects, while the others showed limited or inconsistent influence.

The moderating effect of learner level was generally limited. However, in the context of positive emotions, when examining the effect sizes among various learner stages, secondary students exhibit a more prominent enhancement in learning performance compared to tertiary and mixed groups. This is in accordance with the findings of Camacho-Morles et al. (2021). There are several explanations for the stronger effects observed in secondary students. Firstly, secondary school students are in a critical period of cognitive and emotional development. Their self-identity and emotion management skills are still in the process of formation, making them more susceptible to the influence of emotions. Secondly, younger students generally have a higher level of enthusiasm for learning, while older learners tend to experience a relatively lower level of enjoyment (Vierhaus et al., 2016). The reduced enjoyment among older students may lead to less variability in positive emotions, potentially resulting in weaker correlation magnitudes within these samples. Future empirical studies should incorporate elementary and middle school students to explore the potential influence of learner level on the relationship between positive emotions and learning performance. Regarding negative emotions, upon comparing the effect sizes across different learner stages, it is evident that mixed and tertiary students display a more pronounced improvement in learning performance than secondary students. This finding is consistent with that of Teimouri et al. (2019).

Discipline did not significantly moderate the overall relationship between achievement emotions and learning performance. However, subgroup trends suggested some differentiation. For positive emotions, comparing effect sizes across disciplines reveals that the improvement in learning performance is more pronounced in humanities and social sciences than in natural sciences, differing from the findings of Camacho-Morles et al. (2021). Regarding negative emotions, the impact of enhancing students’ learning performance is more prominent in the domain of natural sciences in comparison to that of humanities and social sciences. The factors contributing to these outcomes might potentially stem from the discrepancies in discipline classification norms. The discipline classification scheme utilized in this study is relatively extensive and lacks sufficient refinement. It could also be attributed to the restricted sample sizes or the divergences in the learning performance metrics that were applied in previous investigations within the natural sciences. These outcomes thereby signify the exigency for further research endeavors to identify more illustrative and reliable indicators of academic achievement.

The type of online learning modality did not significantly moderate the overall relationship between achievement emotions and learning performance. Nonetheless, effect sizes differed across modalities. When comparing the effect values of distinct online learning styles, it is discerned that the enhancement of students’ learning performance is more conspicuous in blended learning and online synchronous learning than in online asynchronous learning. This finding aligns with the results reported by Deri et al. (2024). The reason may be that when learning synchronously, students can engage in real-time interaction and communication with teachers and peers, receiving immediate feedback and support. Meanwhile, in synchronous learning, students feel more sense of social presence, which leads to more intense impact. Compared to synchronous learning, asynchronous learning offers students greater autonomy and flexibility (Wu et al., 2023). However, the absence of immediate feedback and interaction may diminish the impact of achievement emotions on students’ learning performance.

The type of technology significantly moderated the relationship between achievement emotions and learning performance, particularly for negative emotions. A comparison of effect sizes shows that the enhancement of students’ learning performance is more pronounced in mixed and efficiency-oriented technologies than in communication and collaboration technologies. Efficiency-oriented technologies, which focus primarily on improving learning efficiency and effectiveness, provide direct support for students’ learning tasks, thereby bolstering their sense of achievement and self-efficacy. In contrast, communication and collaboration tools—such as discussion forums, chat platforms, or peer-feedback systems—demonstrated relatively weaker effects. One possible explanation is that these tools, while enabling interaction, may fail to evoke strong achievement emotions without adequate instructional scaffolding. Without clear structure or instructor presence, learners may experience superficial engagement or feel emotionally unsupported. This suggests that platform design should incorporate affective scaffolding, such as emotionally responsive prompts, structured dialog frames, or AI-assisted facilitation. Moreover, instructor presence remains critical—teachers need to monitor, guide, and emotionally anchor peer interactions to maximize the motivational and cognitive benefits of social technologies.

These findings carry actionable implications for practice. First, instructors should foreground efficiency-oriented features to establish early mastery experiences, then embed social technologies within clearly structured, emotionally supportive tasks. For example, brief adaptive quizzes can precede a forum discussion, allowing students to approach peer interaction with heightened confidence and enjoyment. Moreover, communication tools should incorporate emotion-responsive prompts—such as periodic self-reflection check-ins or guided sentence starters—to channel peer exchanges toward constructive, motivating dialog. Second, platform designers are encouraged to integrate emotion-aware affordances: conversational agents that deliver just-in-time encouragement when sentiment analysis detects anxiety or boredom, dialog frames that require learners to elaborate before posting, and instructor dashboards that visualize aggregate emotional trends so teachers can intervene when negative affect rises.

Type of performance measure significantly moderated the relationship between achievement emotions and learning performance. Notably, the association was somewhat stronger when learning performance was assessed through self-reported measures compared to standardized tests, consistent with prior meta-analytic evidence (Camacho-Morles et al., 2021). One possible explanation is that self-reported measures, often administered during the learning process, may be more sensitive to students’ momentary emotional and motivational states. These measures frequently capture constructs such as self-efficacy, engagement, and satisfaction, which are themselves closely tied to emotions and have established links to performance outcomes. In contrast, standardized tests—typically administered at fixed time points—may underrepresent the dynamic interplay between affect and learning, potentially attenuating observed associations. While statistically significant, the modest effect size indicates that performance measure type is only one of several interacting factors, and its influence should be interpreted within a broader contextual framework.

From a practical standpoint, periodic low-stakes affective check-ins—such as brief confidence or enjoyment scales embedded after each instructional segment—can furnish teachers with an emotion-sensitive barometer of learning progress and enable timely intervention before negative feelings erode achievement. For platform designers, incorporating lightweight in-platform self-assessment widgets and visualizing their outputs alongside objective quiz scores in a unified dashboard would allow instructors to triangulate affective signals with summative evidence, thereby obtaining a more comprehensive picture of student progress. Although measure type matters, its moderating effect is modest; combining emotion-sensitive self-reports with rigorous summative tests will give the most balanced picture of how achievement emotions influence performance in real-world online courses.

## 6 Conclusions and future directions

This meta-analysis comprehensively examined the relationship between achievement emotions and students’ learning performance in online learning environments. The findings strongly support the conclusion that achievement emotions play a significant role in influencing learning performance, suggesting that positive emotions can improve performance, while negative emotions can hinder performance. Interestingly, the beneficial effects of positive emotions appear stronger among secondary school students, while the adverse impact of negative emotions becomes more pronounced with age. Moderator analyses indicate that the correlations between positive emotions and learning performance do not significantly differ across learner levels, academic disciplines, online learning styles, or technology types, but do vary significantly according to the type of performance measure used. Similarly, negative emotions show stable effects across most learner and instructional variables, yet demonstrate significant variability when moderated by technology types and performance measures. Distinct from prior meta-analyses that examined traditional or blended learning environments, this study focuses exclusively on fully online environments and systematically investigates how various contextual moderators shape the relationship between achievement emotions and learning performance in digital education. These findings suggest that educators and policymakers should place greater emphasis on addressing students’ achievement emotions to better support their learning performance.

This meta-analysis makes a remarkable contribution to the comprehension of achievement emotions within the educational domain, covering both theoretical and practical dimensions. Theoretically, this study extends and augments the extant literature regarding achievement emotions and learning performance, demonstrating that achievement emotions represent a significant determinant in enhancing students’ learning outcomes. From a practical vantage point, the analysis of moderating effects discloses that various elements, including learner level, discipline, online learning style, and technology type, have an impact on how achievement emotions affect learning performance. Future research should address five key points: (1) Since most studies in the inclusion criteria focus on secondary and tertiary students, it is recommended to include elementary learners to broaden the scope of emotion research. (2) The emotional differences across various academic disciplines suggest that teachers should adjust their instructional strategies based on the characteristics of the subject. In the humanities, teachers can use emotionally rich teaching activities to evoke positive emotions in learners, thereby increasing their engagement. In the natural sciences, teachers should help students better cope with challenges and reduce the interference of negative emotions, such as by guiding students to develop more positive coping strategies and confidence in problem-solving. (3) It shows that combining synchronous and asynchronous learning methods can significantly enhance learners’ positive emotional experiences. Therefore, teachers can leverage synchronous classes to enhance interaction, while providing flexible learning resources and opportunities through asynchronous learning to meet the needs of different students and improve learning performance. (4) The use of educational technology should not only focus on learning performance but also consider its potential impact on emotions. Different technology types have varying effects on students’ emotional regulation. Teachers should select appropriate technology based on instructional goals and student needs, to both improve learning efficiency and optimize students’ emotional experiences. (5) Standardized tests alone may not fully capture the impact of emotions on learning, especially the subtle fluctuations of positive and negative emotions during learning. Various assessment methods, such as self-reporting and systematic automated recording, are recommended, combining quantitative and qualitative data. This allows for a more comprehensive understanding of the impact of emotions on academic performance, leading to more targeted teaching improvements.

This study has several limitations. First, most included studies adopted cross-sectional or correlational designs, limiting causal interpretations. While achievement emotions may influence learning performance, the reverse may also be true. Future research should employ experimental or longitudinal approaches to clarify these relationships. Second, the analysis considered only a limited set of moderators. Variables such as gender, intervention duration, and study design (e.g., cross-sectional vs. longitudinal), noted in prior work (e.g., Botes et al., 2022), were excluded due to data constraints. Future meta-analyses should incorporate these factors to deepen understanding. Third, all studies measured emotions via self-reports, which may introduce bias. As alternative methods (e.g., EEG and heart rate monitoring) become more accessible, future research should adopt multimodal approaches to improve measurement validity.

With the accelerating integration of artificial intelligence into education, future research on achievement emotions in online learning should shift toward understanding emotion–technology interactions in more nuanced ways. A key direction is to explore how AI-mediated feedback influences students’ emotional responses and, in turn, their engagement and performance. AI agents are now common, but we still know little about how their communication style and personalized content affect students’ emotions. Investigating which AI-driven features can effectively mitigate negative emotions and foster positive emotions will be essential for designing emotionally supportive learning environments. Building on this, future studies should examine the potential of AI-assisted emotion regulation strategies in online settings. With advancements in affective computing, it is now feasible to implement real-time emotional monitoring and adaptive interventions. For instance, virtual tutors or conversational agents could be designed to detect emotional cues and deliver timely prompts or scaffolds aimed at sustaining motivation and reducing emotional disengagement. Such approaches represent a promising avenue for enhancing students’ emotional resilience and persistence during autonomous online learning.

## Data Availability

The original contributions presented in the study are included in the article/supplementary material, further inquiries can be directed to the corresponding author.
